# Morbidity Associated with Chronic Hyponatremia

**DOI:** 10.3390/jcm12030978

**Published:** 2023-01-27

**Authors:** Guy Decaux

**Affiliations:** Research Unit for the Study Hydromineral Metabolism, Department of Internal Medicine, Erasmus University Hospital, Molière-Longchamps Hospital, Rue Marconi, 142, B-1190 Brussels, Belgium; guy.decaux@skynet.be

**Keywords:** fall, bone fracture, osteoporosis, sarcopenia, hypercalciuria

## Abstract

This article will discuss the consequences of chronic hyponatremia. In conditions such as cancer, heart failure, liver cirrhosis, or chronic kidney disease, the presence and magnitude of hypotonic hyponatremia are considered to reflect the severity of the underlying disease and are associated with increased morbidity as well as mortality. Hyponatremia can be acute (<48 h) or chronic (>2–3 days). Chronic hyponatremia is associated with attention deficit, dizziness, tiredness, gait disturbance, falls, sarcopenia, bone fractures, osteoporosis, hypercalciuria (in the syndrome of inappropriate antidiuresis—SIADH), and kidney stones. In vitro studies have shown that cells grown in a low concentration of extracellular sodium have a greater proliferation rate and motility. Patients with chronic hyponatremia are more likely to develop cancer. We will not review the clinical consequences of respiratory arrest and osmotic demyelination syndrome (ODS) of the too-late or excessive treatment of hyponatremia.

## 1. Introduction

About 5% of adults have a serum sodium (SNa) of <135 mEq/L, rising to an incidence of 20% in people over 65 years of age, and can reach even 30% in hospitalized patients [1]. In the cases where the etiology of hyponatremia does not appear to be obviously known, serum osmolality measurements can help to rule out possible non-hypotonic hyponatremia (hyperproteinemia, hyperlipemia, and translocational hyponatremia). We will not discuss non-hypotonic hyponatremia here.

In virtually all common conditions, such as heart failure, liver cirrhosis, cancer, or chronic kidney disease, the presence and magnitude of hypotonic hyponatremia are considered to reflect the severity of the underlying disease and are associated with increased morbidity or mortality [1] (see later).

## 2. Symptoms of Acute (<48 h) Hyponatremia (and Complications of Its Treatment)

Patients with severe hyponatremia (usually <115–120 mEq/L) are exposed to serious neurological complications. On the one hand, a rapid decrease in serum sodium can cause brain edema, increasing intracranial pressure because of the rigid confines of the skull. If hyponatremia is insufficiently corrected or remains untreated, uncontrolled seizures, non-cardiogenic pulmonary edema, brain herniation, or cardiorespiratory arrest may develop, with major risks of neuropathological sequelae or death [2,3]. On the other hand, excessive correction induces brain dehydration and could be followed by brain demyelinating lesions (central pontine and extrapontine myelinolysis or ODS) [4,5], but we will not discuss this here [5].

## 3. Asymptomatic Hyponatremia Is Not Really Asymptomatic

We will concentrate on the various well-identified complications for patients with chronic hyponatremia (>48 h).

### 3.1. Mild Chronic Hyponatremia Is Associated with Unsteadiness and Attention Deficits

Mild chronic hyponatremia may be responsible for falls, especially in elderly people, these probably resulting from attention, posture, and gait impairments [6]. In a previous study we selected 16 patients with chronic hyponatremia (mean SNa of 128 ± 3 mEq/L) and considered to be clinically asymptomatic. These patients did not have complaints and had normal neurologic examinations. At the time, there was no hint to either the patient or the physician that indicated the existence of hyponatremia. All patients had hyponatremia due to the syndrome of inappropriate antidiuresis (SIAD). We performed eight different visual and auditory tests using computerized attention tasks. Patients were seated in a quiet and isolated room in front of a 14-inch computer screen at a viewing distance of 40 to 50 cm. The median response time (on different tests) during hyponatremia was 672 ± 182 msec, and after the normalization of serum sodium it was 615 ± 184 msec, with a highly significant difference of 58 msec (*p* < 0.001). Moreover, total errors during hyponatremia were increased 1.2-fold (*p* < 0.001). For comparison, in a study conducted on 10 controls (of similar age and sex) the intake of alcohol (0.55 g/kg body weight) induced an increase in median latency of only 25 msec (*p* = 0.001). It is evident that these patients with supposedly asymptomatic hyponatremia should not be allowed to drive a car.

**Figure 1:**
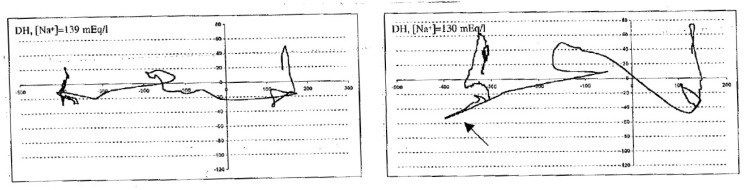
Evolution of the total travelled way (TTW) by the center of pressure in the dynamic test (to walk on the platform three stereotyped steps “in tandem”, eyes open) in a 60-year-old patient with mild asymptomatic hyponatremia and after correction. Patient is walking from right to left.

In the same study mentioned previously, we also analyzed posture and tandem gait in 12 patients (mean age of 61 ± 12 years) with chronic hyponatremia (SNa of 128 ± 3 mEq/L). Posture and tandem gait parameters were investigated with a pressure-sensitive calibrated platform (Footscan; RS Scan International, Olen, Belgium). This platform evaluated the patients’ balance performance (during hyponatremia) based on the recorded displacement of the center of pressure (see Figure 1).

After the normalization of serum sodium, patients were evaluated again. In tandem gait, patients with clinically asymptomatic hyponatremia had large increases in their “total traveled way” (TTW) (from approximately 1 m with normal serum sodium to 1.3 m during hyponatremia). A typical case is presented in Figure 1. As a model of comparison after mild alcohol intake (0.55 g/kg body weight), which is well-known for attention and balance impairment, the TTW in tandem gait increased slightly from approximately 1 to 1.1 msec (*n* = 10) [6].

### 3.2. Epidemiology of Falls, Bone Fractures, and Other Symptoms in Patients with Chronic Hyponatremia

**Figure 2:**
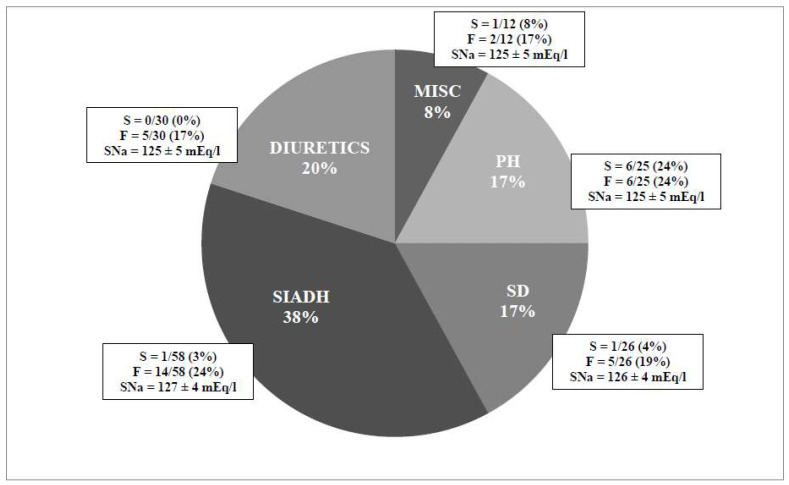
Causes of hyponatremia; number of falls and seizures in 151 consecutive patients without edema or ascites hospitalized via the emergency room. SD: salt depletion; PH: polydypsia; MISC: miscellaneous causes of hyponatremia; S: seizure; F: fall; and SNa: mean serum sodium of the subgroup on admission.

In 151 consecutive hyponatremic patients without edema or ascites (mean SNa of 125 ± 5 mEq/L; mean age of 70 ± 14 years) seen in the emergency room and admitted to the medical ward of a general hospital in Brussels, a fall was given as the reason for admission in approximately 23% of the patients [7]. This study only included patients with asymptomatic hyponatremia between 115 and 132 mEq/L (see Figure 2). In another analysis where we excluded patients with suspected acute hyponatremia (polydipsia hyponatremic syndrome and/or the presence of seizures), we found that the frequency of falls was approximately 21% (see Figure 2) in the 122 remaining patients (mean SNa of 126 ± 6 mEq/L). As expected, these patients were considered clinically asymptomatic by the medical staff of the ward. In contrast, in the control group of 244 consecutive patients with a normal serum sodium level, only 13 (5.3%) reported falls as a major complaint [7]. After controlling for sex, age, and other factors known to be associated with falls (e.g., medication), the adjusted odds ratio for falls in patients with hyponatremia was 67 compared with controls [6]. Interestingly, even very mild hyponatremia was associated with a 20% risk of a fall (in 63 consecutive patients with a serum sodium level between 127 and 132, 22% were admitted for a fall); however, except for falls that were not due to seizures, these patients were considered to be asymptomatic. As expected, the increase in the incidence of falls in patients with asymptomatic hyponatremia is also associated with an excess of bone fractures [8,9,10,11,12,13,14,15]. It has been shown that older people are much more affected by attention and postural balance deficit than in younger adults with mild to moderate chronic hyponatremia [16].

The above being the case, maintaining SNa above 134 mEq/L will likely decrease morbidity associated with falls and attention deficit, especially in elderly patients. In a similar way, the resolution of hyponatremia in the elderly improves neurocognitive (ΔMMSE 1.8 ± 3 vs. 0.7 ± 1.9; *p* < 0.002) and motor performance (ΔADL 14.3 ± 17.1 vs. 9.8 ± 14.7; *p* < 0.002) [17]. Chronic hyponatremia, as in SIAD, is also associated with osteoporosis, which may contribute to bone fractures (see later).

The antiseizure medications oxcarbamazepine (OXC) and carbamazepine (CBZ) are frequently prescribed for the pharmacological treatment of focal epilepsy as well as for trigeminal neuralgia and bipolar disorders, and these medications are known for inducing hyponatremia. A recent article compares the symptoms related to mild hyponatremia with those in normonatremia (see Table 1). Dizziness, tiredness, instability, diplopia, falls, nausea, vomiting, and an increased frequency of seizures were reported more often in the hyponatremic group than in patients with normal serum sodium [18]. On the one hand, the risk of falls can be attributed to impairments in attention, and on the other hand to gait disturbances. Nerve conduction velocity (NCV) is known to be affected by hyponatremia [19]. The slowing in nerve conduction velocity reflects a reversible functional alteration in nerve impulse generation and propagation; given the role of extracellular sodium in this process we believe that the slowing of NCV plays a significant role in gait disturbances, together with the cerebellar ataxia and attention deficits induced by hyponatremia. This has recently been shown in a rat model of SIADH [20]. During the physiological process of aging, a decrease in nerve conduction velocity and sensory amplitude has been well-documented (about 1 m/s per decade) [21]. This could contribute to the greater sensitivity of the elderly to hypotonic hyponatremia, especially in the gait tests (longer axons conducting more slowly [19,20]).

In a case–control study of 513 cases of bone fracture after incidental falls in ambulatory patients aged 65 or older [8], the prevalence of hyponatremia in patients with bone fractures and controls was, respectively, 13% and 3.9%. In this study, hyponatremia was mild (mean SNa of 131 mEq/L) and asymptomatic.

It must be noted that hyponatremia was either drug-induced (36% diuretic, 17% selective serotonin re-uptake inhibitors, and idiopathic SIAD in 37%). Hyponatremia was associated with 9.2% of all bone fractures. Avoiding iatrogenic hyponatremia or treating hyponatremia likely may decrease the number of bone fractures in this population. A recent study shows that correction of hyponatremia in hospitalized patients with SIAD might have a positive impact on osteoblast function [13].

### 3.3. Sarcopenia

Chronic hyponatremia (>1 month) is also associated with a decrease in food intake [22,23] and sarcopenia [23,24,25]. In these studies, patients were included only if it was certain that hyponatremia had existed for at least more than one month (for most of these patients hyponatremia had been present for many months (but not treated)). Gait instability and falls, as described above, could contribute to less physical activity and an ensuing loss in muscle mass (sarcopenia). As a possible consequence, a more sedentary lifestyle could also decrease the need for calorie intake, explaining the lower urine solute output in patients with chronic SIADH [23,25]. We also know that sarcopenia contributes to osteoporosis [26]. In many patients with low creatininuria (reflecting sarcopenia), the long-term treatment of hyponatremia reverses these abnormalities [27].

### 3.4. Osteoporosis and Hypercalciuria

**Figure 3:**
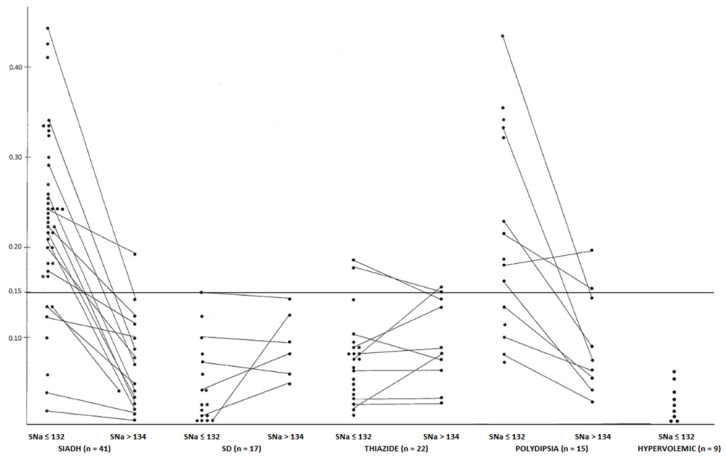
Evolution of the UCa/UCr ratio before and after the correction of hyponatremia of different origins (the horizontal line represents the upper normal limit obtained in adults before breakfast) (adapted from ref. [36] with the permission of the authors).

Chronic hyponatremia is well-known to be associated with osteoporosis and bone fractures [28]. This osteoporosis was first shown in rats [29]. Many factors contribute to this complication. A direct effect of low SNa on in vitro bone composition has been shown to be a result of the activation of the osteoclasts [30]. Chronic hyponatremia is usually associated with an increase in ADH, and this could also stimulate bone resorption as both receptors for V1 and V2 have been described on the osteoclasts [31]. The two AVP receptors, AVPr1α and AVPr2, are expressed in osteoblasts as well as osteoclasts enhancing and reducing, respectively, the formation of bone-resorbing osteoclasts and bone-forming osteoblast [31]. Hypercalciuria is observed in hyponatremia, secondary to SIADH and attributed to the increase in effective volemia [32,33]. In idiopathic hypercalciuria osteoporosis is reported [34], and a recent article has shown that chronic hyponatremia is associated with a higher risk of kidney stones [35]. Figure 3 shows the high calciuria (represented by the UCa/UCr ratio) in patients with hyponatremia secondary to SIAD [36].

### 3.5. Excess Mortality in Chronic Hyponatremia

Hyponatremia of different origins is associated with a significant increase in the length of stay in a hospital, meaning increased costs [37]. Most importantly, it has been shown that hyponatremia is associated with an increased risk of mortality, even in patients with slightly reduced serum sodium concentrations [37,38,39]. Several studies have found that the association between serum sodium concentration and mortality in hospitalized patients is nonlinear. Patients with reduced serum sodium concentration have an increased risk of mortality (around 30–40%) [40,41].

There is also convincing evidence that mortality risk is reduced when hyponatremia improves [42].

### 3.6. Hyponatremia and Cancer

Many studies have shown that overall survival in different cancer types is negatively affected by hyponatremia, whereas the correction of serum sodium has a positive effect on a patient’s outcome [42]. A large retrospective study conducted in a Danish population has shown that chronic hyponatremia is associated with a higher risk of developing a neoplasm [43]. In vitro studies have shown that cells grown in a low concentration of extracellular sodium have greater motility and proliferation, due to a dysregulation in intracellular pathways [44]. In addition, vaptans have also been shown to have antiproliferative properties [45].

## 4. Conclusions

Treating hyponatremia will avoid multiple morbidities, such as unsteadiness, attention deficit, falls, and bone fractures. It will improve neurocognitive and motor performance in the elderly. It will prevent osteoporosis, hypercalciuria, and kidney stones. Treating hyponatremia will decrease the length of hospital stays and decrease hospital costs. There is also convincing evidence that mortality risk is reduced when hyponatremia is corrected. The treatment of hyponatremia associated with cancer will have a positive impact on patient survival.

## Figures and Tables

**Table 1:** Frequency of some symptoms related to hyponatremia between 128 and 134 mEq/L (*n* = 254) compared to patients with normonatremia (SNa > 134 mEq/L) (*n* = 300). All of the patients are treated with carbamazepine (CBZ) or oxycarbamazepine (OXC) (adapted from ref. [13]).

	Na 128–134 (254) (%)	Na > 134 (300) (%)	*p*-Value
Cognitive slowing	18 (7.1)	0	<0.001
Concentration problems	15 (5.9)	4 (1.37)	<0.001
Confusion	8 (3.1)	2 (0.7)	<0.001
Diplopia	33 (13)	12 (4.0)	<0.001
Dizziness	61 (24)	19 (6.30)	<0.001
Falls	16 (6.3)	1 (0.3)	<0.001
Gait disturbance	6 (2.4)	2 (0.7)	0.002
Headache	14 (6.7)	9 (3)	<0.001
Increased seizure frequency	16 (6.3)	0	<0.001
Instability	43 (16.9)	8 (2.7)	<0.001
Nausea/vomiting	14 (5.5)	1 (0.3)	<0.001
Somnolence	21 (8.3)	6 (2)	<0.001
Tiredness	44 (17.3)	20 (6.7)	<0.01

## Data Availability

Not Applicable.
